# Effects of Protective *Lacticaseibacillus casei* VC201 Culture on Late Blowing Prevention, Lipid Profile, and Sensory Characteristics of Valtellina Casera PDO Cheese During Ripening

**DOI:** 10.3390/foods14142433

**Published:** 2025-07-10

**Authors:** Francesca Bonazza, Stefano Morandi, Tiziana Silvetti, Alberto Tamburini, Ivano De Noni, Fabio Masotti, Milena Brasca

**Affiliations:** 1 Institute of Sciences of Food Production (CNR-ISPA), National Research Council, Via Celoria 2, 20133 Milan, Italy; stefano.morandi@cnr.it (S.M.); tiziana.silvetti@cnr.it (T.S.); milena.brasca@cnr.it (M.B.); 2 Department of Agricultural and Environmental Sciences, University of Milan, Via Celoria 2, 20133 Milan, Italy; alberto.tamburini@unimi.it; 3 Department of Food Environmental and Nutritional Sciences, University of Milan, Via Celoria 2, 20133 Milan, Italy; ivano.denoni@unimi.it (I.D.N.); fabio.masotti@unimi.it (F.M.)

**Keywords:** spoilage, bio-protection, clostridia, antimicrobial, cheese

## Abstract

This study aimed to verify, under real operating conditions, the effectiveness of protective lactic acid bacteria (LAB) culture in counteracting the development of late blowing defects in Valtellina Casera PDO cheese and its impact on product sensory characteristics. Thirty-four LAB isolated from Bitto and Valtellina Casera PDO cheeses were screened for anti-*Clostridium* activity. *Lacticaseibacillus casei* VC201 was able to inhibit all the indicator strains through organic acid production. Valtellina Casera PDO cheese-making was performed twice in three dairy farms using a commercial autochthonous starter culture with and without the addition of the protective culture VC201. Cheese was ripened both at 8 °C and 12 °C and analyzed after 70 and 180 days for LAB population, proteolysis, and lipolysis evolution as well as sensory impact. Cheeses with the addition of the VC201 strain showed higher contents of rod-shaped LAB throughout the ripening at both temperatures. The protective culture decreased the production of butyric acid at 70 days, especially at 8 °C (−15.4%), while butyric fermentation was occasionally lightly observed at 12 °C. The sensory profile was favorably impacted by the higher relative proportion of short-chain fatty acids (SCFFAs, C2–C8), which was especially pronounced at 8 °C and persisted for 180-day ripening (23.91% vs. 18.84% at 70 days and 23.84 vs. 21.71 at 180 days of ripening). The temperature and time of ripening had a significant effect on the free fatty acid content of the cheese samples in all three classes (SCFFA, MCFFA, and LCFFA). The cheese made with *Lcb. casei* VC201 was preferred, according to the sensory evaluation, being perceived as less acidic, less bitter, tastier, and with more intense flavor. Protective cultures can represent a practical way to reduce late blowing defects in Valtellina Casera cheese production while maintaining adherence to its PDO regulatory requirements.

## 1. Introduction

Northern Italy’s Sondrio province is the designated area to produce Valtellina Casera, a semi-hard cheese made from milk from local cow breeds mainly fed hay and wild herbs from the restricted area. Produced since around 1500, this cheese was certified as a Protected Designation of Origin (PDO) in 1996. In accordance with the PDO rules, the milk from two or more milkings is partially skimmed and coagulated with calf rennet and naturally occurring microorganisms. The PDO specifications give no indication about heat treatment of the starting milk, which therefore could be processed raw or after pasteurization. To date, about 90% of the total Valtellina Casera production is obtained from pasteurized milk. The curd is progressively pressed for 8 to 12 h after 30 min of cutting at 40 to 45 °C. The maturation procedure takes place for a minimum of 70 days. The final cheese is shaped like a regular cylindrical conformation, measuring 30 to 45 cm in diameter, 8 to 10 cm in height, and weighing between 7 and 12 kg [1].

During the ripening process, complex biochemical reactions occur, which aid in the breakdown of primary constituents and the creation of secondary metabolites, defining the sensory profile. Lipolysis represents an essential pathway to flavor development. The hydrolysis of triacylglycerols by lipolytic enzymes results in the release of free fatty acids (FFAs), which directly affect the sensory attributes of cheese or serve as essential components for additional biochemical alterations [2,3]. These secondary reactions yield volatile and non-volatile compounds, which are essential aroma-active components in cheese that considerably affect its flavor and aroma, including methyl ketones, aldehydes, secondary alcohols, lactones, and esters [4,5]. These biochemical processes interact to produce a wide range of chemical compounds that affect the final flavor, texture, and composition of the final product [6,7,8].

According to Collins et al. (2003) [6], there are six different sources of lipolytic enzymes that aid in cheese ripening: (1) milk, (2) rennet, (3) starter bacteria, (4) microbial secondary cultures, (5) nonstarter lactic acid bacteria (NSLAB) and other ripening microorganisms, and (6) exogenous lipase preparations [4,9,10]. When triglycerides are broken down by lipases, medium- and long-chain fatty acids, partial glycerides, and glycerol are produced [6,7,11]. Cheese flavor development is considerably impacted by FFAs released through lipolysis, primarily short and medium-chain fatty acids (from C4:0 to C8:0 and from C10:0 to C14:0, respectively) [4]. Conversely, because of their high thresholds for perception, long-chain fatty acids (>14 carbon atoms) minimally affect the flavor [12].

The development of holes, cracks, and splits in cheese, frequently linked to rancid flavor and unpleasant odors, is known as the late blowing defect (LBD) [13]. LBD occurs in semi-hard and hard cheeses during the ripening process, and the anaerobic conditions encourage endospore germination [14]. Raw and pasteurized milk hard and semi-hard cheeses, including Beaufor, Edam, Emmental, Gouda, Grana Padano, Gruyère, Kasseri, Parmigiano Reggiano, Provolone, and Queso Manchego, are frequently affected by LBD [13].

While several *Clostridium* species, including *Clostridium beijerinckii*, *Cl. butyricum*, as well as *Cl. sporogenes*, have been connected, it is commonly acknowledged that *Cl. tyrobutyricum* is the main responsible species [15,16]. The production of hydrogen, carbon dioxide, acetic, and butyric acids by clostridia during the breakdown of lactate leads to the development of cavities (also called “eyes”), cracks, and structural defects in cheese [17,18]. Since it depends on variables like cheese variety and age, it is challenging to determine a critical level of butyric acid for the indication of LBD. However, a butyric acid content of more than 100 mg/kg of cheese already showed evidence of butyric acid fermentation in Gouda cheese [19].

Several strategies can be adopted to prevent clostridial spoilage, such as physical treatments like bactofugation and microfiltration [20], as well as the use of additives like lysozyme or nitrate [21]. According to recent studies, ripening conditions that include low temperatures (≤15 °C), a lower pH (<5.00), and osmotic stress can prevent LBD [22]. Despite this existence of several methods, many approaches are not applicable or are expressly forbidden for specific cheese varieties, particularly those that fall under the strict regulatory requirements of PDO cheeses [23]. In this regard, adding protective cultures such as nisin-producing lactic acid bacteria represents an encouraging strategy to limit clostridial growth, reducing the incidence of structural defects in hard and semi-hard cheeses [24,25]. The inhibitory activity against *Clostridium* spore germination, which represents the principal reason for LBD in cheese, has been demonstrated for lactic acid bacteria (LAB), and some experimental trials have shown the efficiency of these bacteria to prevent the defect [16]. *Lactococcus lactis*, *Lacticaseibacillus casei*, and *Lactobacillus delbrueckii* subsp. *bulgaricus* have been reported to exert inhibitory activity against *Cl. tyrobutyricum* through the production of hydrogen peroxide, organic acids, and bacteriocins [26]. The addition of LAB bacteriocins or the incorporation of LAB strains for in situ bacteriocin production could represent a predominant approach. According to a recent investigation, these strains continue to be viable during the ripening process and throughout the cheese manufacturing process [16]. Evidence reported that the antimicrobial mechanism includes (i) microbial interaction, such as competitive exclusion of pathogenic microorganisms through resource utilization [27]; (ii) biosynthesis of specific antimicrobial metabolites with anti-clostridial properties, including bacteriocins [18]; and (iii) modification of the physicochemical parameters affecting the clostridial proliferation [22]. Together, these effects reinforce the LAB’s protective function during cheese ripening, preventing LBD from occurring. Currently, the PDO specification does not regulate the use of starter cultures for the production of Valtellina Casera. The protection consortium has solicited this research to provide scientific support for the request to amend the production rules at the European level in this regard.

The use of LAB strains isolated from the cheese itself, as a result of the selective pressure of the environment and technology applied during cheese-making, may be an effective strategy to counteract the development of LBD [16,24,25,26]. Most studies, following in vitro verification of strain activity, have performed scale-up tests in model cheeses [25] or cheese slurry [24]. In our study, we wanted the scale-up to take place under real production conditions of Valtellina Casera PDO and to involve three different cheese factories to verify the reproducibility of the results.

In this context, the aims of this study were (i) to identify a promising autochthonous LAB strain with anti-clostridial activity and (ii) to verify, under real operating conditions in three different dairy plants, its potential effectiveness in counteracting the development of late blowing defects in Valtellina Casera cheese under two different ripening temperature conditions and its impact on sensory characteristics.

## 2. Materials and Methods

### 2.1. Bacterial Strains and Culture Conditions

This study was carried out on 34 LAB strains (2 *Lb. delbrueckii* subsp. *lactis*, 5 *Lbc. casei*, 18 *Lacticaseibacillus paracasei*, 4 *Lacticaseibacillus rhamnosus* and 5 *Lactiplantibacillus plantarum*) previously isolated from Bitto and Valtellina Casera PDO cheeses [28,29]. Twenty-two clostridial strains from four different species were used as indicator microorganisms to evaluate the LAB antimicrobial activity. These strains were provided by different culture collections (*Cl. beijerinckii* DSM 791^T^, *Cl. butyricum* DSM 10702^T^, *Cl. sporogenes* ATCC 3584^T^, *Cl. sporogenes* ATCC 10000, *Cl. tyrobutyricum* DSM 2637 and *Cl. tyrobutyricum* ATCC 8260) or isolated from cheeses (8 *Cl. sporogenes* and 8 *Cl. tyrobutyricum*) with LBD [30]. Before each experiment, LAB strains were propagated in de Man Rogosa and Sharpe (MRS) broth (Scharlab, Barcelona, Spain) at 30 °C (*Lcb. casei*, *Lcb. paracasei* and *Lpb. plantarum*) or 37 °C (*Lb. delbrueckii* subsp. *lactis Lcb. rhamnosus*) for 24 h, while the *Clostridium* strains were grown anaerobically (AnaeroGen™, Thermo Fisher Scientific, Waltham, MA, USA) in Reinforced Clostridial Medium (RCM) broth (Scharlab) at 37 °C for 72 h. All strains were refreshed twice before testing.

### 2.2. Anti-Clostridium Activity and Safety Assessment

The anticlostridial activity was evaluated by the agar disk diffusion and overlay method. The agar diffusion method was used to make a preliminary assessment of 34 LAB’s antimicrobial activity against the *Clostridium* strains provided by the culture collections. This assay was performed as previously described by Morandi et al. (2025) [30]. The six strains with the best anti-*Clostridium* activity were subsequently tested by the overlay method against *Cl. sporogenes* (9 strains) and *Cl. tyrobutyricum* (9 strains) isolated from dairy products. Briefly, overnight cultures of LAB were inoculated as two 1 cm long lines in MRS agar (Scharlab) plates and incubated at 30 °C for 48 h. The plates were then overlaid with RCM (Scharlab) soft agar (10 mL; 0.8% agar) inoculated with 10^4^ cells of *Clostridium* strains. The plates were incubated (37 °C, for 48 h, anaerobically) and subsequently checked for the zones of inhibition around the bacterial streaks. The overlay method and the result interpretation were carried out according to Magnusson et al. (2003) [31].

To understand the nature of the antimicrobial activity of LAB strains, cultures of the *Lactobacillaceae* family were grown in MRS broth and then centrifuged at 9300× *g* for 5 min (Benchtop centrifuge 5425, Eppendorf, Hamburg, Germany). The cell-free supernatant (CFS) was recovered, sterilized by filtration through a 0.20 μm pore size PDVF membrane filter (Minisart, Sartorius, Goettingen, Germany) and concentrated to 1/10 of their original volume in a vacuum concentrator. Later, different aliquots of CFS were adjusted to pH 6.0 with sterilized 1 N NaOH and were subjected to the following treatments: (i) catalase digestion (1 mg/mL; Sigma-Aldrich, St. Louis, MO, USA) at 37 °C for 1 h, to eliminate the possible inhibitory action of hydrogen peroxide; (ii) proteinase K digestion (1 mg/mL; Sigma-Aldrich) at 37 °C for 2 h, to evaluate the production of proteinaceous antimicrobial compounds; (iii) heating at 60, 100 °C for 60 min, and at 121 °C for 15 min. Another CFS aliquot was separately used for detecting the lysozyme production by LAB strains. The antimicrobial activity of different CFS aliquots was monitored by the agar disk diffusion method as described before. Lastly, lysozyme-like protein production was assayed in MRS agar with the addition of lyophilized cells of *Micrococcus lysodeikticus* ATCC 4698 (10% w/w) (Sigma-Aldrich). Hen egg-white lysozyme (Sacco S.r.L., Cadorago, Italy) was used as the positive control according to Maidment et al. (2009) [32].

LAB strains were also evaluated for their antibiotic susceptibility considering the microbiological cut-off values indicated by EFSA guidelines regarding microorganisms used as feed additives or as production organisms [33] as previously described by Morandi et al. (2024) [34]. The strain meeting safety requirements and expressing the strongest anti-*Clostridium* activity was chosen for the experimental production of Valtellina Casera cheese.

### 2.3. Autochthonous Anti-Clostridium Culture Preparation

The protective culture was preliminary grown two times in UHT milk at 30 °C for 24 h and then used to inoculate a suitable volume of milk that was finally employed for the experimental cheese-making trials. Each experimental cheese-making was carried out using approximately 5 L of milk starter culture being added at a 1% level. For this purpose, the protective culture was prepared in a single fermenter (30 °C per 18 h), distributed under refrigerated conditions to the dairy farms involved in the study, and used in cheese-making within 24 h of its production. An aliquot of protective culture was collected for microbiological analysis before distribution.

### 2.4. Cheese Manufacture and Sampling

Valtellina Casera cheese-making was performed with pasteurized milk with the addition of a commercial autochthonous starter culture composed by *Lc. lactis* subsp. *lactis*, *Lc. lactis* subsp. *cremoris*, *Streptococcus thermophilus*, *Lactobacillus delbrueckii* subsp. *bulgaricus*, and *Lactobacillus helveticus* (Lyofast SLH 071 CB, Sacco srl) in three dairy farms (A, B, C) of Valtellina valley following the PDO regulation [1]. A total of 48 wheels (16 for each dairy) were considered in this study, 24 obtained with the addition of the protective culture selected in this study (Protective Starter PRO) and the other 24 without the protective strain (commercial autochthonous starter, CAS). All cheese-making was simultaneously prepared from the same bulk milk. Summarizing, each dairy farm produced 8 Valtellina Casera wheels made with the addition of protective culture and 8 cheese wheels without the protective strain. At the end of the cheese-making processes, the cheeses from the three farms were collected and all ripened in the same ripening room at 8 °C (24 wheels) and 12 °C (24 wheels) for 180 days.

A quarter wheel of Valtellina Casera was collected at different ripening periods (70 and 180 days) and immediately transferred to the laboratory under refrigerated conditions. Each cheese sample was cut and divided into wedges approximately 3 cm thick. Sampling was conducted at 3 cm from both the upper and lower rinds and 1.5 cm from the heel. Microbiological analyses were performed within 24 h of sample arrival. For chemical analysis, a slice of cheese representative of the whole wheel was ground and collected in polyethylene bags, stored at −18 °C and thawed at 20 °C before analysis.

External indicators of LBD (cheese swelling and cracks) were monitored throughout the ripening period of the cheeses, while symptoms of LBD in the cheese matrix (irregular eyes, cracks/splits and unpleasant odors) were recorded at each sampling time.

### 2.5. Microbiological Analysis

To analyze the content of the protective LAB strain in the starter, one millilitre of culture was serially diluted in strength Ringer’s solution (Scharlab) and inoculated into MRS agar (Scharlab). The plates were incubated under anaerobic conditions (AnaerocultA, Merck, Darmstad, Germany) at 30 °C for 72 h. To evaluate the LAB population in the cheese, ten grams of samples were homogenized in 90 mL of a 2% (w/v) sterile K_2_HPO_4_ buffer solution (Sigma-Aldrich) for 2 min in a Stomacher BagMixer (Interscience, St. Nom, France). Samples were serially diluted in quarter-strength Ringer’s solution (Scharlab) and inoculated into the following culture media: MRS agar (Scharlab) acidified with acetic acid to pH 5.4 under anaerobic conditions (Anaerocult A) at 30 °C for 72 h for mesophilic rod LAB; M17 agar (Biolife Italiana; Milan, Italy) at 37 °C for 48 h for coccus LAB; Kanamycin Aesculin Azide (KAA) agar (Scharlau) at 37 °C for 48 h for Enterococci; Petrifilm Lactic Acid Bacteria Count Plates (3M, Minneapolis, MN, USA) at 30 °C for 48 h for heterofermentative LAB.

### 2.6. Chemical Analysis

A central slice (approx. 200 g) of the cylindrical cheese wheel was cut, finely ground, and used for chemical analyses. Dry matter, fat, and protein were determined according to ISO methods [35,36,37], as described in Masotti et al. (2023) [38]. The soluble nitrogen (SN) at pH 4.4 (pH-4.4 SN) was measured by the Kjeldahl method as described in standard [39] using a K-439 automatic digestor and a K-375 distillation and titration unit (Büchi Labortechnick, Flawil, Switzerland). All analyses were carried out in triplicate.

The pH 4.4 insoluble fractions of the cheese samples were also measured by urea–polyacrylamide gel electrophoresis (urea-PAGE) performed on a Hoefer vertical slab gel unit according to the method proposed by Andrews et al. (1983) [40] with modifications by Veloso et al. (2004) [41] on a 7.5% resolving gel. Electrophoresis was performed on a mini vertical electrophoresis unit (SE250, Hoefer, Holliston, MA, USA) at a constant voltage of 90 V, as described by Masotti et al. (2023) [38].

### 2.7. Free Fatty Acid Extraction and Analysis

Free fatty acids (FFAs) were extracted following the protocol described by [42]. In brief, 5.0 g of cheese sample was homogenized with anhydrous Na_2_SO_4_ (J.T.Baker, Deventer, The Netherlands) and acidified with H_2_SO_4_ (Supelco, Merck KGaA, Darmstadt, Germania) (0.2 mL). Before extraction, 1.0 mL of internal standard solution consisting of pentanoic acid (C5:0) (Fisher Scientific, Bishop Meadow Road, England, UK), nonanoic acid (C9:0) (Fisher Scientific), tridecanoic acid (C13:0) (Fisher Scientific), and heptadecanoic acid (C17:0) (Sigma Aldrich, Merck KGaA, Darmstadt, Germania) in heptane (Supelco) was added to perform quantitative analysis. Solid-phase extraction (SPE) was conducted using amino-modified silica gel cartridges (Chromabond NH_2_, Macherey-Nagel, Düren, Germany) to isolate FFAs from neutral lipids. The SPE cartridges were activated with heptane, followed by the elution of lipophilic compounds using a diethyl ether (Supelco) /heptane solution (1:1, v/v). Subsequent washing was performed with a chloroform/isopropanol (Sigma Aldrich) solution (2:1, v/v), and FFAs were eluted using a 2% formic acid (Sigma Aldrich) in diethyl ether solution (Supelco).

An Agilent 6890 gas chromatography system (Agilent Technologies, Santa Clara, CA, USA) was employed for free fatty acid quantification, equipped with an autosampler, on-column injector, and flame ionization detector (FID). Analytes were introduced via cool on-column injection, with the detector maintained at 275 °C. Chromatographic separation was achieved using a Stablewax-DA capillary column (Restek Corporation, Bellefonte, PA, USA; 15 m × 0.53 mm internal diameter, 1 μm film thickness) with hydrogen as the carrier gas. The thermal gradient program consisted of a starting temperature of 65 °C, followed by a linear temperature ramp of 10 °C min^−1^ to 240 °C, concluding with 12.5 min of an isothermal period. Chromatographic data acquisition and peak integration were performed using MassHunter B.07.04.2260 software (Agilent Technologies). Qualitative identification of FFAs was performed through retention time comparison with authenticated reference standards (Supelco and Restek Corporation, Bellefonte, PA, USA). Quantitative results were calculated and expressed as mg FFA per 100 g of cheese. To observe the different distribution of FFA groups, the acidic profile of the experimental cheeses was also studied.

### 2.8. Sensory Analysis

A triangle test was implemented to evaluate the impact of the addition of the protective culture on the sensory characteristics of the cheese after 110 days of ripening. The external part of the cheese wheel (approximately 1 cm) was removed, and sticks (1.5 × 1.5 × 6 cm) were portioned and put at 20 °C one hour before tasting. Three randomly coded samples—two were the same and one was different—were presented in equal quantity to the panelists. Panelists (n = 18; 11 male; 7 female; aged 30–65 years) were untrained, frequent consumers of cheese that were asked to evaluate the samples in the order in which they were presented and to identify the sample which differed from the others, giving reasons for their choice. Even if unsure, participants were requested to choose one sample as different. The order of samples was balanced and theses were distributed randomly in groups of six. Water and unsalted crackers were available for mouth rinsing. The test organization and significance of the results were evaluated according to the ISO standard [43]. A significant difference between the samples was considered when the correct responses were higher than 10 (expected for 18 panelists with a *p* value of 0.05).

### 2.9. Statistical Analysis

Data were analyzed using SAS (version 9.4; SAS Institute Inc., Cary, NC, USA). The results obtained for plate counts were transformed to log10 for statistical analysis to obtain a normal distribution of the residuals in linear statistical models. Data were analyzed by the GLM procedure, with a model of 3 class factors (starter culture, temperature of ripening, and ripening period) with interactions.

## 3. Results and Discussion

### 3.1. Screening for LAB Strains with Antimicrobial Activity Against Clostridia

Thirty-four LAB isolated from Bitto and Valtellina Casera PDO cheeses were screened for anti-*Clostridium* activity against *Cl. beijerinckii* DSM 791^T^, *Cl. butyricum* DSM 10702^T^, *Cl. sporogenes* ATCC 3584^T^, and *Cl. tyrobutyricum* IN15b. The primary screening was performed by the agar disk diffusion method, and six strains (*Lb. delbrueckii* subsp. *lactis* VC108, *Lcb. casei* BT147 and VC201, *Lcb. paracasei* BT202 and VC213, and *Lcb. rhamnosus* VC220) showed a high antimicrobial activity. These LAB were then selected for further analysis to evaluate their ability to inhibit the growth of 18 *Clostridium* strains belonging to *Cl. sporogenes* (n = 9) and *Cl. tyrobutyricum* (n = 9) isolated from dairy products affected by LBD (Table 1). Two strains of *Lcb. casei* were able to inhibit all the indicator strains; in particular, VC201 showed a strong antimicrobial activity (growth inhibition from >4% of plate area per bacterial streak) against 5 *Cl. sporogenes* and *4 Cl. tyrobutyricum* strains (Table 1). These findings agreed with Trevisol et al. (2024) [16], who reported that eight *Lcb. casei* strains were able to inhibit the development of *Clostridium* spp., and their activity was comparable to the lysozyme used in LBD prevention.

To investigate the nature of the anti-*Clostridium* activity, the CFS of *Lcb. casei* VC201 was submitted to different treatments (neutralization, catalase and protease K digestion, and heat treatments). Only pH neutralization eliminated the CFS antimicrobial activity, highlighting that the capability of *Lcb. casei* VC201 to inhibit the *Clostridium* growth was due to organic acid production. Moreover, the result of the lysozyme-like protein detection test on MRS agar revealed that this strain was not able to produce this enzyme. Organic acids, usually lactic or acetic acid (but also others including formic, citric, succinic, glutamic, and phenyllactic), are responsible for the CFS antimicrobial activity of many LAB strains. These compounds, as undissociated acids, can penetrate the cytoplasmic membrane of target microorganisms, thus resulting in intracellular acidification interfering with ATP, RNA, and protein synthesis; DNA replication; and in the collapse of the transmembrane proton motive force [44]. The antimicrobial activity of organic acids is not only related to pH reduction but also to other mechanisms; for instance, lactic acid can permeabilize the outer membrane of Gram-negative species, causing structural alterations in the phospholipid component and consequently cell death [45]. Although in our study we did not investigate in detail which organic acids are produced by *Lbc. casei* VC201 in cheese, a study by Zalan et al., 2010 [46] showed that strains of *Lbc. casei*, after growing in milk, in addition to lactic acid, also produced acetic acid and succinic acid, which are characterized by marked anti-clostridic activity [47].

*Lcb. casei* VC201 was found to be sensitive to all tested antibiotics and for this reason was chosen as the protective culture in the following cheese-making trials.

**Table 1:** Antimicrobial activity of selected LAB strains against *Cl. sporogenes* (9 strains) and *C. tyrobutyricum* (9 strains) isolated from dairy products with LBD. Result interpretation was performed according to Magnusson et al. (2003) [31].

LAB Strains	*Cl. sporogenes* (9 Strains)				*Cl. tyrobutyricum* (9 Strains)				Total (%)	
	-	+	++	+++	-	+	++	+++	++	+++
*Lb. delbrueckii*										
VC208	6	3	-	-	4	5	-	-	0.0	0.0
*Lcb. casei*										
BT147	-	-	4	5	-	4	2	3	33.4	44.4
VC201	-	-	4	5	-	1	4	4	44.4	50.0
*Lcb. paracasei*										
BT202	7	1	1	-	5	4	-	-	5.6	0.0
VC213	5	3	1	-	5	1	3	-	22.2	0.0
*Lcb. rhamnosus*										
VC220	2	2	4	1	1	3	5	-	50.0	5.6

-: no inhibition. +: weak inhibition (antimicrobial activity from 0.1 to 2% of the plate area per bacterial streak). ++: moderate inhibition (antimicrobial activity from 2 to 4% of plate area per bacterial streak). +++: strong inhibition (antimicrobial activity from >4% of plate area per bacterial streak). Magnusson et al. (2003) [31].

### 3.2. Statistical Analysis

The experimental cultures CAS and PRO were used to produce Valtellina Casera PDO cheese, which was ripened in two different conditions (8–12 °C) and matured for 70 and 180 days. Although the replication of the experimental tests in three different dairies resulted in a wide variability of results, the analysis of the data highlights some scientific evidence.

The concentration of FFAs in the cheese samples was significantly impacted by the ripening temperature (rT), ripening time (R), and culture CxrT according to the GLM (Table 2), while the microbial population was significantly affected by the ripening time (R) and CxrT. The culture type (CAS vs. PRO) that was employed, on the other hand, had a less significant effect, despite helping to modify some interactions.

**Table 2:** The impact of ripening time and culture type on the chemical and microbiological properties of cheese samples. Data represent the mean ± standard deviation (SD) of 12 samples for each treatment group (CAS and PRO cultures), evaluated after 70 and 180 days of ripening at 8 °C and 12 °C. Statistical analysis considered the following factors: rT = ripening temperature; C = culture; R = ripening time. Significance levels are indicated as follows: *** *p* < 0.001, ** *p* < 0.01, * *p* < 0.05, and † *p* < 0.10.

	RIPENING TIME (Days)																				
	70								180												
	Culture								Culture												
	CAS				PRO				CAS				PRO								
	rT (°C)				rT (°C)				rT (°C)				rT (°C)								
	8		12		8		12		8		12		8		12		Probability				
Variables	Mean	SD	Mean	SD	Mean	SD	Mean	SD	Mean	SD	Mean	SD	Mean	SD	Mean	SD	rT	C	CxR	CxrT	RxrT
Enterococci_log10	4.62	0.52	5.10	1.19	5.08	1.06	5.39	1.07	3.83	0.82	3.16	0.47	2.98	0.27	3.19	0.97					
Heterofermentative_log10	4.29	0.52	3.95	0.00	4.29	0.52	4.18	0.55	2.95	0.00	2.96	0.02	2.95	0.00	2.95	0.00					
Cocci_LAB_log10	6.93	0.77	7.56	0.51	8.01	1.03	8.45	0.48	6.54	0.42	6.47	0.93	5.84	1.02	6.24	0.72			***		
Rods_LAB_log10	6.35	0.61	7.29	0.97	8.14	1.08	8.35	0.6	6.04	1.23	6.82	0.97	8.24	0.18	7.93	0.54	†	***			
Dry matter (%)	63.25	2.60	64.84	3.37	63.22	2.47	64.98	3.07	66.12	2.65	66.84	3.15	66.44	2.01	67.14	2.71					
Protein (%)	26.24	1.62	27.30	2.17	26.31	1.44	27.32	1.79	28.34	1.30	29.08	1.57	28.48	1.06	29.35	1.59	†				
pH 4.4-SN (% TN)	12.47	0.91	14.80	1.28	12.38	1.22	15.4	1.80	19.08	1.42	21.93	2.12	17.63	2.61	21.35	2.17	***				
Fat in dry matter (%)	46.67	2.15	45.60	2.52	46.19	1.80	46.39	1.45	47.47	2.20	47.24	2.80	46.74	2.26	47.49	1.84					
FFAs (mg/100 g of cheese)																					
C2:0	6.16	1.02	7.97	1.77	7.02	1.07	8.32	4.00	6.77	1.42	11.32	3.30	8.11	2.55	10.92	4.44	**				
C3:0	0.26	0.07	0.52	0.35	0.30	0.10	0.23	0.07	0.24	0.17	1.18	1.24	0.15	0.05	1.24	2.11	*				†
C4:0	2.20	0.20	3.40	1.03	1.86	0.43	3.30	2.02	3.34	0.63	15.74	10.46	3.39	0.78	15.5	16.03	**				**
C6:0	0.89	0.14	1.18	0.24	0.77	0.29	1.30	0.40	1.03	0.56	5.32	1.46	1.52	0.72	4.35	0.71	***				***
C8:0	0.38	0.20	0.80	0.11	0.24	0.11	0.80	0.11	0.39	0.13	3.39	0.97	0.42	0.15	2.64	0.57	***				***
C10:0	1.35	0.69	2.81	0.20	0.89	0.44	2.73	0.32	1.20	0.32	8.11	2.38	1.28	0.4	6.35	0.98	***				***
C10:1	0.15	0.08	0.31	0.03	0.10	0.05	0.30	0.06	0.14	0.03	1.05	0.31	0.15	0.04	0.82	0.16	***				***
C12:0	2.00	0.87	3.25	0.27	1.17	0.80	3.25	0.38	1.67	0.42	9.73	2.33	1.80	0.56	7.52	0.96	***				***
C12:1c	0.01	0.00	0.02	0.01	0.01	0.01	0.03	0.03	0.02	0.01	0.04	0.01	0.02	0.01	0.03	0.01	**				
Ci14:0	0.05	0.03	0.08	0.02	0.02	0.02	0.06	0.02	0.04	0.02	0.21	0.06	0.04	0.01	0.21	0.07	***				***
C14:0	4.38	1.85	8.55	0.72	3.22	1.23	8.21	1.07	4.33	1.03	23.56	7.52	4.39	1.14	18.46	3.01	***				***
C15:0	0.41	0.19	0.79	0.09	0.32	0.13	0.82	0.12	0.42	0.10	2.45	0.82	0.39	0.08	2.02	0.33	***				***
C16:0	15.25	5.29	28.93	4.70	12.01	3.33	27.89	3.31	14.42	2.62	75.93	15.86	15.97	4.63	59.76	9.78	***			†	***
C16:1	1.92	0.67	3.84	0.70	1.50	0.56	3.57	0.55	1.86	0.22	9.12	0.84	2.43	1.07	7.06	1.10	***			*	***
C18:0	4.67	1.17	7.61	2.26	3.80	0.83	7.04	0.83	3.92	0.72	18.64	5.48	4.13	0.73	14.91	2.23	***				***
C18:1c9	12.99	4.59	25.07	2.61	9.10	2.94	21.63	2.45	13.00	2.79	73.49	25.18	11.51	3.41	57.85	7.33	***				***
C18:2	1.84	0.78	3.49	0.72	1.39	0.47	3.19	0.34	1.84	0.21	10.85	2.45	2.08	0.45	7.07	2.46	***			*	***
C18:3	0.14	0.08	0.36	0.08	0.10	0.04	0.34	0.05	0.16	0.06	1.22	0.85	0.12	0.03	1.09	0.36	***				***
C18:2c9t11	0.10	0.04	0.22	0.07	0.07	0.03	0.21	0.05	0.10	0.03	0.67	0.49	0.08	0.04	0.62	0.22	***				***

### 3.3. LAB Population in Experimental Valtellina Casera PDO Cheese During Ripening

Table 3 summarizes the LAB content observed during the Valtellina Casera ripening period. Different experimental conditions did not significantly alter microbial populations, especially enterococci and heterofermentative LAB (Table 2). The coccus-shaped LAB count determined in M17 agar after 70 days of ripening differed among samples and was lower in the cheeses obtained without the addition of protective culture at the two temperature conditions (8 °C and 12 °C). Nevertheless, the content of these microorganisms reached the same level after 180 days of ripening at both temperatures (Table 2). Likewise, cheese made with the addition of *Lb. reuteri* to prevent the LBD showed significantly lower lactococcus counts than the control cheese [48]. A negative effect of *Lcb. casei* on the growth of *S. thermophilus* and *Lb. delbrueckii* subsp. *bulgaricus* species, present in the starter used in cheese-making, has already been reported by other authors [49] and invites further investigation into the dynamics of this interaction.

As expected, the use of the protective culture led to a greater content of lactobacilli in all the experimental samples, with higher differences at 8 °C, and these values remained constant during the ripening period. This result supports what was reported in a recent study that investigated the efficacy of multiple strains of *Lbc. casei* in counteracting the LBD caused by clostridia in Montasio PDO cheese, where the *Lbc. casei* remained between 10^8^ and 10^9^ cfu/g throughout the ripening process (120 days) [16].

In the cheeses obtained with the addition of the VC201 strain, the ripening temperature did not affect the lactobacillus load, while differences of about 1 log were observed in the control samples (Table 1 and Table 3).

**Table 3:** LAB enumeration results in different media at various stages of Valtellina Casera PDO ripening.

Ripening	Sample	M17 (cocci LAB)		MRS pH 5.4 (rods LAB)	
Temperature (°C)		70 days	180 days	70 days	180 days
8	CAS	6.9 ± 0.7	6.3 ± 0.6	6.3 ± 0.5	6.0 ± 0.8
	PRO	7.9 ± 0.9 ^A^	5.8 ± 0.9 ^B^	8.0 ± 0.9	8.3 ± 0.5
12	CAS	7.2 ± 0.5	6.5 ± 0.8	7.7 ± 1.0	7.0 ± 0.9
	PRO	8.5 ± 0.4 ^A^	6.3 ± 0.6 ^B^	8.4 ± 0.2	8.0 ± 0.5
Probability RT		0.097	0.496	0.094	0.599
Probability Sample		0.0001	0.0001	0.003	0.143

CAS: cheese obtained without the addition of protective culture. PRO: cheese made with the addition of protective culture. Data are expressed as means (data expressed in log10 cfu/g; mean values of two determinations ± Standard Deviation). A, B Means with different letters within a row are significantly different (*p* < 0.05).

### 3.4. Chemical Composition and Proteolysis of Valtellina Casera PDO Samples

The compositional properties of the Valtellina Casera samples were studied to evaluate the effect of the three experimental factors under investigation, i.e., the type of starter (CAS and PRO), the storage temperature (8 °C and 12 °C), and the storage time (70 and 180 days). Few data are available in the literature on the compositional parameters of Valtellina Casera. Mean protein levels were in line with data reported by Salvadori et al. (1999) [50], whereas only a slightly lower fat content was observed (46,8% vs. 50–55% on dry matter). Dehydration of the cheese is one of the processes involved in ripening, which promotes compositional changes, as reported in Table 2. The compositional parameters were unaffected by the type of starter culture, as shown in Table 2. The pH 4 soluble nitrogen (SN) fraction represents an accepted index of the degree of primary proteolysis. As expected, higher values were found at 12 °C, indicating increased proteolytic activity under these conditions. As reported in Table 2, temperature had a moderate impact on protein content. Ripening temperature affects cheese maturation. Higher temperatures generally accelerate the ripening process, leading to faster proteolysis [51]. Data are summarized in Table S1, which reports the gross composition and pH 4.4 soluble nitrogen (pH 4.4-SN) values of Valtellina Casera cheese samples manufactured with (PRO) and without (CAS) *Lcb. casei* VC201.

**Figure 1:**
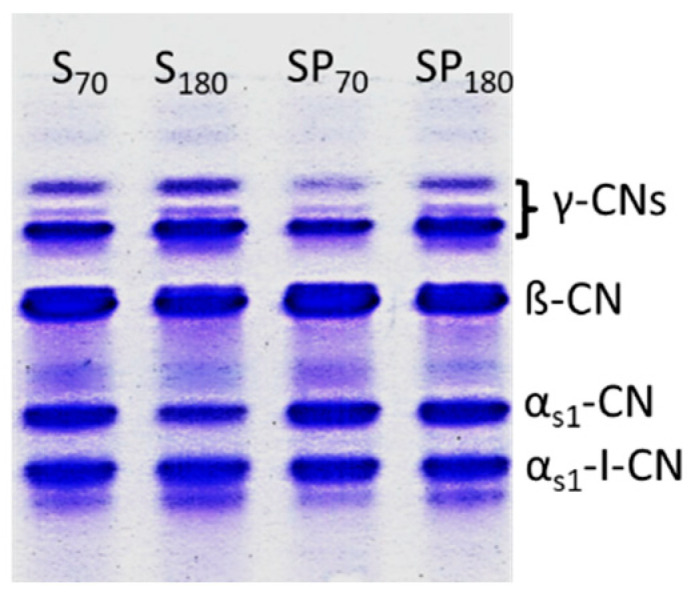
Urea–polyacrylamide gel electrophoretograms of Valtellina Casera cheeses ripened at 12 °C. S: Valtellina Casera sample manufactured with CAS starter; SP: Valtellina Casera sample manufactured with PRO starter. Lower case numbers are storage days.

The electropherograms obtained by urea-PAGE highlighted some differences in the band densities of main casein fractions and their main degradation products throughout storage, whereas no visible effects were attributable to the use of two different starters (Figure 1). Main casein fractions as well as new-formed peptides were identified by comparison with the data from the scientific literature [52].

The patterns of S (containing CAS starter) and SP (containing PRO starter) samples were similar and characterized for dense bands of the major CN fractions (α_s1_-CN and β-CN) and degradation products (i.e., α_s1_-I-CN and γ-CNs peptides). In particular, the γ-CN fractions, deriving from the plasmin-associated hydrolysis of β-CN, slightly increased during ripening. Differently, the intensities of α_s1_-I-CN peptide were similar in all samples.

### 3.5. Free Fatty Acid (FFA) Content in Valtellina Casera PDO Cheese

Even if several studies [53,54] show that the addition of secondary cultures such as *Lb. casei* can stimulate lipolysis, differently than expected, in our study, the total content of FFAs given by lipolysis is greater in cheese where VC201 is missing.

As reported in Table 4, the total FFA content increases with ripening and shows higher values in CAS cheeses than in PRO samples where a protective culture was added, with some exceptions. Greater lipolysis is observed in cheeses produced with a starter culture alone. Notably, the addition of a protective culture has a different impact on cheese lipolysis in relation to the temperature adopted during cheese ripening, showing a higher increase in FFAs throughout ripening at 8 °C.

After 70 days at 8 °C ripening conditions, the average concentrations of total FFAs ranged from 41.25 to 67.94 mg/100 g in CAS cheese and 35.55 to 56.68 mg/100 g in PRO counterparts.

**Figure 2:**
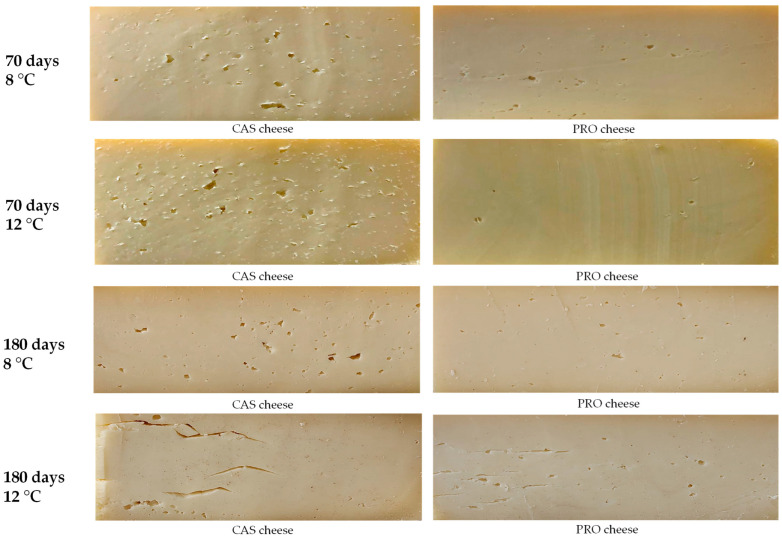
Comparison of eye formation in transverse sections of Valtellina Casera PDO cheese produced using an autochthonous commercial culture (CAS) and a protective culture (PRO), evaluated after 70 and 180 days of ripening at two ripening temperatures (8 °C and 12 °C).

Conversely, after 180 days, significant increases were noted at 12 °C, indicating elevated enzymatic activity during the maturation period (Table 4). The differences between the cheese made with the two starter cultures were more evident; across all dairies, CAS had a greater FFA concentration with a value range after 180 days between 221 mg/100 g and 316 mg/100 g, compared to PRO, which showed a range of 205 to 226 mg/100 g, as also clearly reflected in the eye formation shown in Figure 2.

**Figure 3:**
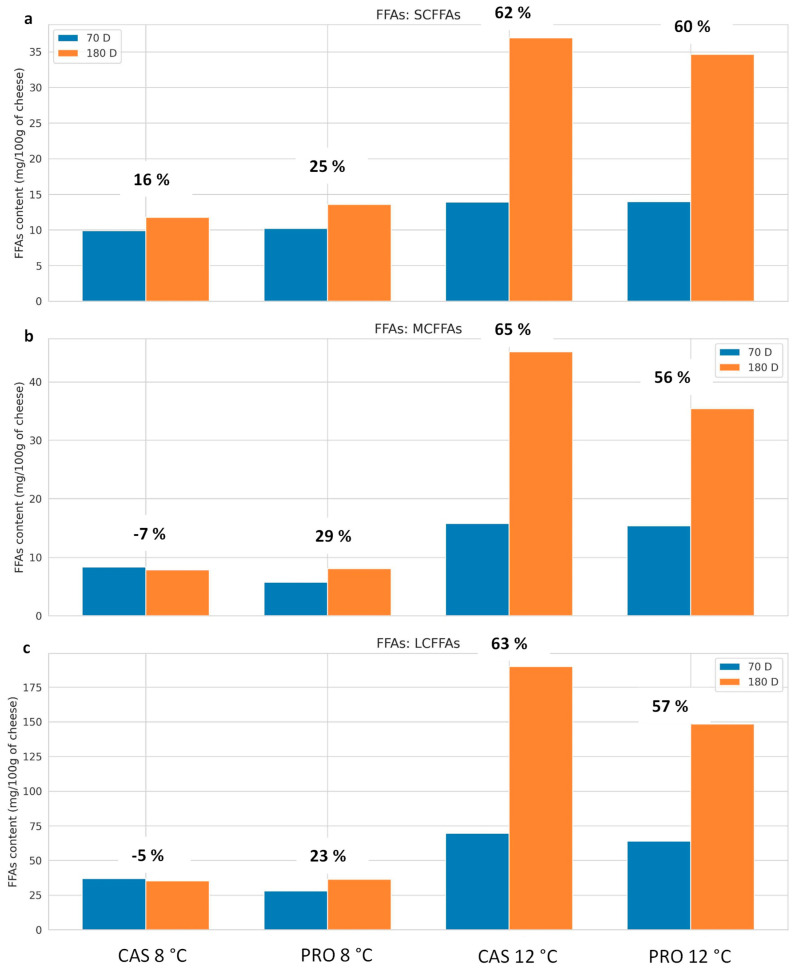
Temperature and ripening time impact on FFA classes: (**a**) SCFFA (short-chain free fatty acid), (**b**) MCFFA (medium-chain free fatty acid), (**c**) LCFFA (long-chain free fatty acid), and Δ%.

As reported by other authors [55,56,57], temperature had a marked impact on lipolytic activity for all fatty acid groups (Table 2). During cheese ripening, lipases from milk and microorganisms are more likely to release SCFFAs, which are the primary contributors to aroma development [6,58,59]. As reported in Table 2, with elevated levels at 12 °C, SCFFAs (C2:0, C3:0, and C4:0) exhibited moderately significant effects (*p* < 0.01–0.05), mostly in response to rT. Significantly, the RxrT interaction also had an impact on C4:0 and C6:0, which increased at 12 °C during the ripening. The interaction of RxrT was significant for many FFAs, indicating that higher temperatures increase the effect of maturation. In experimental cheeses, SCFFAs C2-C8 were already higher after 70 days in PRO cheese (9.42–10.64 mg/100 g) than in CAS cheese (8.84–10.51 mg/100 g) when ripening occurred at 8 °C, showing an increase from 70 to 180 days (Table 4 and Figure 3a). Differently, cheeses produced without the VC201 adjunct culture were characterized by low and stable SCFFA levels at 8 °C, whereas at 12 °C, they showed higher values compared to PRO cheeses as ripening progressed (180 days).

**Table 4:** Mean free fatty acid classes concentrations (mg/100 g) in Valtellina Casera PDO cheese manufactured with autochthonous commercial starter (CAS) and with the addition of protective culture (PRO) at 70 and 180 days of ripening at storage temperatures of 8 °C and 12 °C. (SCFFA (short-chain free fatty acid), MCFFA (medium-chain free fatty acid), LCFFA (long-chain free fatty acid)).

Dairy	Ripening Temperature (°C)	Ripening Time (Days)	Culture	FFAs Mean Values (mg/100 g)			
				SCFFA	MCFFA	LCFFA	Total
A	8	70	CAS	10.51 ± 1.28	9.26 ± 0.95	36.42 ± 2.00	56.18 ± 1.67
			PRO	10.64 ± 3.18	8.21 ± 3.24	37.83 ± 0.95	56.68 ± 7.37
		180	CAS	11.90 ± 1.50	9.52 ± 0.11	39.66 ± 1.01	61.09 ± 2.62
			PRO	12.33 ± 1.90	8.92 ± 3.16	36.96 ± 13.93	58.22 ± 18.99
B	8	70	CAS	8.84± 0.58	11.25 ± 4.22	47.84 ± 17.41	67.94 ± 22.21
			PRO	9.42 ± 1.20	4.11 ± 0.27	22.01± 0.04	35.55 ± 1.52
		180	CAS	11.13 ± 1.61	5.64 ± 1.42	30.10 ± 5.52	46.88 ± 5.33
			PRO	14.65 ± 3.92	7.77 ± 2.64	39.07 ± 15.50	61.43 ± 22.07
C	8	70	CAS	10.30 ± 1.19	4.51 ± 0.29	26.43 ± 2.75	41.25 ± 1.27
			PRO	10.52 ± 0.20	4.88 ± 0.64	24.10 ± 3.16	39.50 ± 4.00
		180	CAS	12.27 ± 2.22	8.32 ± 0.46	36.11 ± 0.74	56.70 ± 1.02
			PRO	13.78 ± 2.59	7.52 ± 2.26	33.01 ± 6.77	54.32 ± 11.62
A	12	70	CAS	16.38 ± 3.77	16.10 ± 1.41	70.34 ± 6.30	102.82 ± 3.95
			PRO	14.12 ± 5.20	15.48 ± 3.66	62.55 ± 11.01	92.16 ± 19.88
		180	CAS	49.28 ± 17.27	45.74 ± 9.50	183.96 ± 72.98	279.00 ± 99.75
			PRO	48.82 ± 31.04	33.47 ± 6.84	142.40 ± 12.99	224.70 ± 50.87
B	12	70	CAS	11.75 ± 0.24	14.83 ± 0.95	64.71 ± 4.69	91.30 ± 5.87
			PRO	13.81 ± 6.78	14.58 ± 0.52	63.09 ± 4.47	91.49 ± 2.83
		180	CAS	25.11 ± 2.15	33.63 ± 0.74	162.45 ± 4.50	221.20 ± 5.90
			PRO	25.62 ± 12.20	33.49 ± 3.93	145.85 ± 13.34	204.96 ± 29.47
C	12	70	CAS	13.52 ± 2.77	16.48 ± 0.11	73.50 ± 2.66	103.51 ± 0.0
			PRO	13.93 ± 2.99	16.15 ± 0.33	65.95 ± 0.81	96.04 ± 4.13
		180	CAS	36.46 ± 14.03	56.04 ± 16.20	223.34 ± 39.59	315.84 ± 69.82
			PRO	29.52 ± 1.51	39.25 ± 5.20	156.79 ± 28.49	225.57 ± 32.18
Probability				SCFFA	MCFFA	LCFFA	Total
Dairy				0.104	0.104	0.517	0.333
Culture				0.992	0.028	0.022	0.061
Ripening Temperature				0.000	0.000	0.000	0.000
Ripening Time				0.000	0.000	0.000	0.000
Dairy × Culture × Ripening Temperature × Ripening time				0.170	0.000	0.000	0.000

Among SCFFAs, butyric acid (C4:0) can be adopted as a marker of butyric fermentation related to clostridia growth in cheese [17,60]. Notably, the lowest C4:0 levels were observed in samples produced with a PRO culture ripened at 8 °C for up to 70 days, compared to those treated with the CAS culture. As ripening progressed and temperature rose, the divergence decreased, which indicated a gradual decrease in the protective culture’s inhibitory impact against clostridia. As reported in Table 4, samples ripened at 8 °C showed varied concentration ranging from 1.99 to 2.53 mg/100 g, lower compared to those ripened at 12 °C where variable but generally higher contents were detected, ranging from 0.68 to 6.57 mg/100 g, comparable with semi-hard and semi-cooked curd cheeses subjected to LBD [61].

Samples treated with the two different cultures at 12 °C did not display any relevant differences that could be attributed to the use of the protective VC201 culture. During ripening, C4:0 concentration tends to increase substantially under these conditions, with variations among samples. Temperatures higher than 10 °C have been reported to enhance the proliferation of clostridia implicated in the LBD of Valtellina Casera PDO cheese [22].

It is beneficial to prevent the LBD in cheese by detecting fermentative phenomena that can damage the product, which is made possible by the presence of caproic acid (C6:0). According to Mayenobe et al. (1986) [61], the C4/C6 ratio can be used as an index of blowing risk. When the C4/C6 ratio reaches 4.00, butyric fermentation takes place in cheese without a risk of late blowing defects, and the risk rises in direct proportion to the ratio’s elevation. To this end, C4/C6 ratios were evaluated, confirming the tendency observed for butyric acid content. The lowest ratios were observed in Valtellina Casera PDO samples ripened for 70 days at 8 °C, while values >4 were generally observed under other conditions. As reported in Table 5, after 180 days of ripening at 8 °C, only two CAS samples with ratios of C4/C6 >4 were observed. As shown in Table 5, the C4/C6 ratio tended to increase at a ripening temperature of 12 °C, with high values even after only 70 days. Higher values were also related with longer ripening time. However, the most pronounced effect was observed at 12 °C and 180 days of maturation.

**Table 5:** C4:0 and C6:0 FFA content and C4/C6 ratio in Valtellina Casera DOP cheese by dairy, ripening temperature (8 °C or 12 °C), time (70 or 180 days), and starter culture (with addition of protective culture (PRO) and autochthonous commercial culture (CAS).

Dairy	Culture	Ripening Temperature (°C)	Ripening Time (days)	C4:0 (mg/100 g)	C6:0 (mg/100 g)	C4/C6
A	CAS	8	70	2.21	1.07	2.06
				2.04	0.94	2.17
	PRO			1.55	0.73	2.11
				2.37	1.18	2.01
B	CAS	8	70	1.99	0.73	2.71
				2.14	0.84	2.54
	PRO			1.44	0.44	3.24
				1.45	0.45	3.21
C	CAS	8	70	2.53	0.75	3.36
				2.29	1.00	2.28
	PRO			2.17	0.99	2.20
				2.21	0.80	2.75
A	CAS	8	180	3.24	1.65	1.96
				2.79	1.21	2.30
	PRO			2.28	1.17	1.95
				4.07	2.69	1.51
B	CAS	8	180	2.97	0.87	3.40
				2.81	0.49	**5.72**
	PRO			3.40	1.13	3.02
				2.80	0.86	3.25
C	CAS	8	180	4.00	0.33	**11.98**
				4.23	1.60	2.64
	PRO			4.39	2.11	2.07
				3.42	1.13	3.02
A	CAS	12	70	3.66	1.51	2.42
				5.04	1.07	**4.69**
	PRO			2.03	0.83	2.43
				6.57	1.27	**5.19**
B	CAS	12	70	2.82	0.98	2.86
				2.22	0.89	2.48
	PRO			0.68	1.20	0.56
				2.77	1.03	2.68
C	CAS	12	70	3.98	1.30	3.06
				2.72	1.33	2.04
	PRO			4.20	1.97	2.14
				3.58	1.50	2.39
A	CAS	12	180	35.18	6.57	**5.36**
				18.29	4.05	**4.52**
	PRO			11.33	3.37	3.36
				48.08	4.07	**11.80**
B	CAS	12	180	8.43	4.16	2.03
				7.88	4.23	1.86
	PRO			8.45	4.94	1.71
				6.72	3.78	1.78
C	CAS	12	180	15.86	7.56	2.10
				8.83	5.34	1.65
	PRO			8.77	4.77	1.84
				9.68	5.16	1.88

Ratios of C4/C6 greater than 4 are shown in bold.

An increase in eye formation that appears wider and is accompanied by cracks is linked to a higher C4/C6 ratio. Figure 2 shows a transverse section of Valtellina Casera PDO cheese for both CAS and PRO cheese under two different conditions (8–12 °C) at two ripening periods (70–180 days). The assessment of the cheese wheels, which was conducted with the help of the “Consorzio per la Tutela dei formaggi Valtellina Casera e Bitto” revealed that both experimental cheeses at 180 days of ripening had larger eyes and cracks as the ripening temperature increased (Figure 3). This finding can be attributed to the fact that temperatures above 10 °C and pH increasing as the ripening progresses have been reported to promote the rapid development of clostridia with gas production [22]. Generally, after 70 days of ripening, there are no evident eyes or cracks (Figure 2). The cheese paste has the characteristic fine and diffuse eye formation of this type of cheese [62], except for two CAS cheese samples that showed symptoms of butyric fermentation for the first time of ripening. However, the results of experimental trials among dairies led to wide variability, likely attributable to technological differences and the peculiar environmental microbiome associated with each factory.

As reported in Table 2, MCFFAs (C8:0, C10:0, and C14:0) exhibited notable variations based on rT and the RxrT interaction, with concentrations rising throughout extended ripening (180 days), especially in cheeses made using the CAS culture at 12 °C. Considering medium-chain free fatty acids (MCFFA, C10:0–C15:0), a similar tendency was observed after 70 days of ripening in both experimental studies, with significant increases observed when the temperature was raised from 8 °C to 12 °C. MCFFA values at 12 °C ranged from 14.83 to 16.48 mg/100 g for CAS and 14.58 to 16.10 mg/100 g for PRO, considerably higher than their corresponding values at 8 °C, where values reach 4.51–11.25 mg/100 g for CAS and 4.11–8.21 mg/100 g for PRO.

As reported in Table 2, the RxrT interaction had a significant effect on long-chain free fatty acids (LCFFAs), but C12:1c was an exception, exhibiting no perceptible variation related to RxrT. C16:1 and C18:2 (linoleic acid, LA) showed significant CxrT interactions, with higher concentrations in CAS cheeses that were ripened at 12 °C. The LCFFAs were found to have the most significant temperature effect, with the highest content at both 8 and 12 °C. After 70 days at 8 °C, the mean content was 36.90 mg/100 g for CAS and 27.98 mg/100 g for PRO. At 12 °C, elevated values of 69.52 mg/100 g for CAS and 63.87 mg/100 g for PRO were observed.

To gain a more comprehensive understanding of the lipolytic process over the ripening period, the percentage variation (Δ%) in FFAs was also evaluated. The Δ% in FFA content between 70 and 180 days emphasizes a stronger influence of temperature and also an effect attributable to the starter culture. As reported in Table 5 and Figure 3a, the increase in SCFFAs is more pronounced at 12 °C (+60–62%), indicating, as expected, that higher temperatures stimulate lipolysis [56,57,63]. At 8 °C, PRO showed a greater increase than CAS (+25% vs. +16%), as a consequence of higher lipolytic activity at lower ripening temperatures. In MCFFAs, the impact of temperature was more evident. A strong increase (+56–65%) was observed at 12 °C, while only the PRO strain increased (+29%) at 8 °C, indicating better adaptive capacity of this strain at lower temperatures (Figure 3b). Only the PRO strain increased at 8 °C (+23%), whereas both cultures responded favorably to LCFFAs at 12 °C (Figure 3c).

Since the only difference between the two cultures was the presence of *Lcb. casei* in the PRO culture, which is used for its bioprotective role against clostridia, it was expected that the cheese composition would be minimally affected. However, statistical analysis (Table 2) confirmed that the main effect of culture (C) was rarely significant, and some relevant CxrT interactions emerged, particularly for C16:1 and C18:2. The presence of *Lcb. casei* may cause an indirect decrease in the amount of linoleic acid, adopting the enzyme linoleate isomerase to change it into conjugated linoleic acid (CLA). As a result, the final product contained more CLA and less linoleic acid [64], even though the variation in CLA content among cultures was not statistically significant (*p* = 0.932).

#### Free Fatty Acid (FFAs) Profile in Valtellina Casera PDO Cheese During Ripening

The relative distribution of the different classes of FFAs contributes to defining the overall acidic profile with a direct impact on the characteristics of Valtellina Casera PDO cheese.

The FFA profile during cheese ripening showed differences between the two starter cultures concerning ripening temperature and time. The PRO culture enhanced a higher relative content of SCFFAs C2–C8, particularly at 8 °C (Table 6). The average CAS values of SCFFAs after 70 days of ripening were 18.84% and 23.91% for the PRO culture. These compounds had a relevant impact on the sensory qualities, affecting the aromatic profile [58,59,65]. Therefore, the presence of a higher content of SCFFAs in PRO than CAS cheese could be the reason for the more pronounced and appreciated flavor that emerged from the sensory test. As reported in Table 6, the similarities between the two cultures at 12 °C (PRO: 14.83%, CAS: 13.99%) are primarily due to enzymatic or microbial activity, which tends to yield uniform the fatty acid profiles at that temperature, as reported in the investigation by Sihufe et al. (2007) [56].

A distinct trend is shown in MCFFA (C10–C15) distribution. At 8 °C, CAS cheese exhibited a higher percentage (14.39%) compared to PRO cheese (12.71%), while at 12 °C, the differences were less marked, with constant values for both cultures around 16–16.50%. According to this tendency, the PRO culture appears to be more temperature-sensitive. Although there were no evident differences between the two cultures, they both showed an increase in the long-chain free fatty acid (LCFFA ≥ C16) fraction at 12 °C. CAS samples generally showed slightly higher percentages than PRO samples at 8 °C, with values stable up to 180 days of ripening.

**Table 6:** Free fatty acid distribution (%) during ripening of Valtellina Casera PDO cheese manufactured with an autochthonous commercial culture (CAS) or with the addition of protective culture (PRO). SCFFA: short-chain free fatty acid; MCFFA: medium-chain free fatty acid; LCFFA: long-chain free fatty acid.

Culture	Temperature (°C)	Time (days)	SCFFA (%)	MCFFA (%)	LCFFA (%)
CAS	8	70	18.84 ± 5.29	14.39 ± 3.54	66.75 ± 2.81
		180	21.71 ± 2.33	14.07 ± 1.91	64.2 ± 0.66
	12	70	13.99 ± 1.75	15.93 ± 0.30	70.07 ± 1.50
		180	13.45 ± 3.64	16.56 ± 1.23	69.98 ± 4.11
PRO	8	70	23.91 ± 4.64	12.71 ± 1.38	63.36 ± 3.36
		180	23.84 ± 1.85	13.88 ± 1.30	62.27 ± 1.24
	12	70	14.83 ± 0.33	16.5 ±0.50	68.65 ± 0.45
		180	15.38 ± 4.63	16.24 ± 1.25	68.37 ± 3.62
Probability					
Ripening Temperature			0.992	0.028	0.022
Culture			0.000	0.000	0.000
Ripening Time			0.000	0.000	0.000
Culture × Ripening Temperature × Ripening time			0.150	0.000	0.000

### 3.6. Sensory Effects

A sensory analysis by means of a triangle test was carried out on the experimental cheeses of the three dairies produced with and without the addition of the protective culture after 110 days of aging. The aim of the test was to determine whether there was a perceptible difference between the two experimental cheeses. Three coded samples, two identical and one different, were presented at the same time to the panelists with the task of identifying the different one and, if possible, justifying the choice.

The panel of trained judges (18 members) was able to distinguish, at a significant level of α = 0.001, the two types of cheeses under examination, showing a clear preference for those obtained with the addition of the *Lcb. casei* strain. Cheese produced with the PRO culture and ripened at 8 °C was described as less acidic and bitter, exhibiting enhanced taste and more intense flavor. At 12 °C, PRO cheese was perceived as less bitter compared to that produced with the CAS culture.

## 4. Conclusions

The use of specific adjunct cultures can influence the evolution of the pro-technological microbial population, specifically lactic acid bacteria in the different forms and also microorganisms responsible for the occurrence of anomalous eyes, such as butyric clostridia contributing to the definition of the sensory characteristics of the cheese. A decisive parameter is the rT of the cheese, which concurs with the starter in promoting the development and the metabolic activity of certain microbial groups. The microbiological composition of Valtellina Casera PDO was significantly influenced by the type of culture and ripening time, while the concentration of free fatty acids was significantly affected by both ripening temperature and ripening time. In our study, VC201 addition to the primary starter evidenced a higher relative proportion of short-chain fatty acids (SCFFAs, C2–C8) and marked protective activity against butyric clostridia when cheese was ripened at 8 °C up to 180 days of ripening, while non-effective prevention was observed at 12 °C, temperature that favors the rapid development of butyric clostridia. Sensory activity showed a positive contribution of *Lbc. casei* VC201 in the cheese characteristics at both ripening temperatures, which can be explained by the higher production and proportion of SCFFAs under all operating conditions (times and temperatures) applied in this study.

To use this protective culture in a more effective way in Valtellina Casera production and also in other semi-hard cheeses, optimal ripening conditions should be adopted (e.g., ≤10 °C). Controlled use of *Lbc. casei* VC201 requires further in-depth studies on the co-culture dynamics of *Lbc. casei* VC201 in relation to the LAB species/biotypes used in the primary starter. Metabolomic profile analysis is required for better insight into the organic acids that are the basis of clostridia inhibition activity and to the conditions and factors that favor or limit their production.

## Data Availability

The original contributions presented in the study are included in the article/Supplementary Material, further inquiries can be directed to the corresponding author.

## Supplementary Materials

The following supporting information can be downloaded at: https://www.mdpi.com/article/10.3390/foods14142433/s1. Table S1. Gross composition and pH 4.4 soluble nitrogen (pH 4.4-SN) of Valtellina Casera cheese samples. Valtellina Casera samples manufactured with *Lcb. casei* VC201 (PRO) and without (CAS); SN: soluble nitrogen; pH4.4-SN: SN at pH 4.4.
