# Supplementary material for: Effects of Protective Lacticaseibacillus casei VC201 Culture on Late Blowing Prevention, Lipid Profile, and Sensory Characteristics of Valtellina Casera PDO Cheese During Ripening
Source: Foods. 2025 Jul 10;14(14):2433. doi: 10.3390/foods14142433 (PMC12294488; doi:10.3390/foods14142433)
Supplement: Supplementary file 1 [file foods-14-02433-s001.zip › foods-3712157-supplementary.pdf]

Table S1. Gross composition and pH 4.4 soluble nitrogen (pH 4.4-SN) of Valtellina Casera cheese samples. Valtellina Casera samples manufactured with *Lcb. casei* VC201 (PRO) and without (CAS); SN: soluble nitrogen; pH4.4-SN: SN at pH 4.4.

	CHEMICAL PARAMETERS						
**STARTER**	Ripening temperature (°C)	Storage time (days)	Dry matter (%)	Protein (%)	Fat (%)	FDM (%)	pH 4.4-SN (% TN)
**CAS**	8	70	59.7	24.7	29.1	48.8	12.7
			60.2	24.3	29.7	49.4	12.1
			64.5	27.9	29.1	45.1	13.0
			65.0	26.5	30.0	46.2	10.8
			65.1	28.2	28.5	43.8	13.3
			65.1	25.8	30.4	46.7	12.9
		180	62.7	26.7	31.4	50.1	18.1
			62.8	26.9	31.0	49.4	18.4
			67.2	29.6	30.2	44.9	21.4
			67.5	29.8	33.0	48.9	20.3
			68.2	28.5	31.5	46.2	18.4
			68.3	28.5	31.9	46.7	17.9
	12	70	60.1	24.5	29.3	48.7	13.7
			61.0	24.8	29.0	47.5	13.6
			66.4	27.8	31.0	46.7	16.1
			66.5	28.9	28.4	42.7	15.9
			67.2	28.1	30.5	45.4	13.6
			67.8	29.7	28.9	42.6	15.9
		180	62.8	27.1	31.5	50.2	19.4
			62.9	27.3	31.9	50.7	19.3
			68.5	30.5	29.9	43.6	22.1
			68.5	30.8	30.8	45.0	23.8
			68.6	29.4	32.0	46.6	22.9
			69.8	29.4	33.0	47.3	24.1
**PRO**	8	70	59.4	24.6	28.9	48.6	12.1
			60.8	24.8	29.1	47.9	10.9
			64.3	27.8	28.1	43.7	12.6
			64.8	28.0	29.2	45.1	13.7
			65.0	26.1	30.1	46.3	13.8
			65.1	26.6	29.7	45.7	11.2
		180	63.8	26.8	32.2	50.5	14.5
			64.2	27.8	30.6	47.6	14.2
			66.8	29.6	29.7	44.5	19.7
			67.2	28.6	31.8	47.3	18.4
			67.8	29.5	30.2	44.5	20.1
			68.8	28.6	31.7	46.1	18.9
	12	70	60.9	25.1	28.8	47.2	13.4
			61.1	25.2	29.8	48.8	12.8
			66.8	28.0	30.2	45.2	16.5
			66.8	27.8	31.2	46.7	16.7
			66.9	29.6	30.2	45.1	16.8
			67.3	28.2	30.5	45.3	16.2

180	63.2	27.1	31.5	49.8	20.1
	64.2	27.8	31.8	49.5	18.5
	68.3	30.5	31.5	46.1	21.4
	68.5	31.1	31.6	46.1	20.6
	69.2	29.4	33.0	47.7	22.8
	69.4	30.2	31.7	45.6	24.7
